# Cangfudaotan decoction inhibits mitochondria-dependent apoptosis of granulosa cells in rats with polycystic ovarian syndrome

**DOI:** 10.3389/fendo.2022.962154

**Published:** 2022-11-09

**Authors:** Xiao-lin Jiang, He Tai, Xuan-si Xiao, Shi-yu Zhang, Shi-chao Cui, Shu-bo Qi, Dan-dan Hu, Li-na Zhang, Jin-song Kuang, Xian-sheng Meng, Shun-min Li

**Affiliations:** ^1^ Department of Nephrology, The Fourth of Affiliated Hospital of Guangzhou University of Traditional Chinese Medicine (Shenzhen Traditional Chinese Medicine Hospital), Guangzhou University of Traditional Chinese Medicine, Shenzhen, China; ^2^ Key Laboratory of Ministry of Education for Traditional Chinese Medicine Viscera-State Theory and Applications, Liaoning University of Traditional Chinese Medicine, Shenyang, China; ^3^ College of Pharmacy, Liaoning University of Traditional Chinese Medicine, Dalian, China; ^4^ Department of Internal Medicine, Liaoning Provincial Corps Hospital of Chinese People’s Armed Police Forces, Shenyang, China; ^5^ Science and Technology Branch, Liaoning University of Traditional Chinese Medicine, Shenyang, China; ^6^ NHC Key Laboratory of Male Reproduction and Genetics, Guangdong Provincial Reproductive Science Institute (Guangdong Provincial Fertility Hospital), Guangzhou, China; ^7^ Department of Internal Medicine, Fujian Provincial Corps Hospital of Chinese People’s Armed Police Forces, Fuzhou, China; ^8^ Department of Endocrinology and Metabolism, The Fourth People’s Hospital of Shenyang, Shenyang, China

**Keywords:** polycystic ovary syndrome, granulosa cell, insulin resistance, mitochondrial dysfunction, cangfu daotan decoction, apoptosis

## Abstract

Polycystic ovary syndrome (PCOS) is a universal endocrine and metabolic disorder prevalent in reproductive aged women. PCOS is often accompanied with insulin resistance (IR) which is an essential pathological factor. Although there is no known cure for PCOS, cangfudaotan (CFDT) decoction is widely used for the treatment of PCOS; nevertheless, the underlying mechanism is not clear. In this study, 40 Sprague-Dawley (SD) rats (female) were randomized to 4 groups, namely the control group, PCOS group, PCOS+CFDT group, and PCOS+metformin group. The rats in the control group were fed a normal-fat diet, intraperitoneally injected with 0.5% carboxymethyl cellulose (CMC, 1 mL/kg/d) for 21 days and orally given saline (1 mL/kg/d) for the next 4 weeks. The rats in the PCOS group, PCOS+CFDT group, and PCOS+Metformin group were fed a high-fat diet (HFD) and intraperitoneally injected with letrozole (1.0 mg/kg) for 21 days. During this period, we recorded the body weight, estrous cycles, and rate of pregnancy in all rats. We also observed the ovarian ultrastructure. Blood glucose indices, serum hormones, and inflammatory factors were also recorded. Then, we detected apoptotic and mitochondrial function, and observed mitochondria in ovarian granular cells by transmission electron microscopy. We also detected genes of ASK1/JNK pathway at mRNA and protein levels. The results showed that CFDT alleviated pathohistological damnification and apoptosis in PCOS rat model. In addition, CFDT improved ovarian function, reduced inflammatory response, inhibited apoptosis of granular cells, and inhibited the operation of ASK1/JNK pathway. These findings demonstrate the occurrence of ovary mitochondrial dysfunction and granular cell apoptosis in PCOS. CFDT can relieve mitochondria-dependent apoptosis by inhibiting the ASK1/JNK pathway in PCOS rats.

## Introduction

Polycystic ovarian syndrome (PCOS) is a widespread endocrine and metabolic disorder, prevalent in reproductive aged women. In general, PCOS is characterized by hyperandrogenism, irregular menstrual cycle, abnormal ovarian function, follicular dysplasia (with multiple cystic ovarian follicles), and insulin resistance (IR) (1). PCOS is associated with IR in approximately 75% of cases (2). Hyperandrogenism, IR, hyperinsulinemia, and a variety of endocrine signals in the follicle can disturb follicular activation and growth in women with PCOS. These effects lead to the accumulation of small follicles around the ovary, polycystic morphology, and injury of follicular maturation, thereby leading to anovulation and infertility. As the main feature, IR refers to reduced insulin sensitivity. IR is a hallmark of metabolic dysfunction in patients with PCOS, and is considered a promoter of hyperandrogenism and chronic oligo- or anovulation (3, 4). Additionally, obesity-related inflammation may have potential implications for ovarian physiology due to the dysregulated adipokine secretion, thereby affecting insulin sensitivity. In adolescents with obesity, increased visceral adiposity is also associated with hormonal changes that impair the hypothalamus and the pituitary function and directly affect ovarian function (5, 6).

Metabolic abnormalities, chronic inflammation, and oxidative stress (OS) have been reported to be associated with IR in PCOS, and those features are related to mitochondrial dysfunction in PCOS (7, 8). Mitochondria are the critical energy controller and the fundamental source of cellular reactive oxygen species (ROS). Mitochondrial abnormalities have organism-wide manifestations that can lead to different metabolic disorders (9). Mitochondria are critical organelles for modulating OS. The abnormal mitochondrial genes and mitochondrial abnormalities in PCOS have been studied (10). Thus, it is essential to understand the mechanism of mitochondrial abnormalities in the pathogenesis of PCOS. However, the regulatory mechanism between mitochondrial abnormalities and PCOS is still unclear. Although there is no known cure for PCOS, traditional Chinese medicine (TCM) may offer a specific therapeutic effects for treating PCOS (11).

The Cang-fu-dao-tan (CFDT) decoction is a classic prescription of TCM for the treatment of PCOS. CFDT decoction is one of the most common prescriptions for PCOS patients in East Asian countries (12). According to TCM theories, the leading causes of PCOS-IR include turbid phlegm, blood stasis, stagnation of liver Qi, deficiency of kidney essence, and deficiency of spleen and kidney. The CFDT decoction comprises 16 medicinal herbs, aiming to solve the pathogenesis of phlegm dampness. A clinical study has reported that the CFDT decoction, used alone or in combination with other western medications, can be used in the treatment of women with PCOS (12), however the mechanism of action of this treatment remains unknown (11–13).

## Materials and methods

### Chemicals and reagents

The CFDT decoction (Beijing Kang-Ren-Tang Pharmaceutical Co., Ltd., Chengdu, China) was dissolved in heated (60°C) deionized water to obtain a 3.0 g/mL stock solution and stored at 4°C. Metformin and letrozole were purchased from Solarbio Life Sciences Co., Ltd. (China).

### Chemical component analysis of the CFDT decoction

The CFDT decoction contained Cyperus rotundus (Dried radix of *Cyperus rotundus L*, Xiangfu in Chinese, 10 g), Citrus × aurantium L (Dried peel of *Citrus*, Chenpi in Chinese, 6 g), Atractylodes lancea (Thunb.) DC (Dried stem of *Atractylodes lancea*, Cangzhu in Chinese, 15 g), Pinellia ternata (Dried radix of *Pinellia ternata* (Thunb.) Breit., Banxia in Chinese, 9 g), Wolfiporia cocos (Dried sclerotium of Syagrus romanzoffiana (Cham.) Glassman, Fuling in Chinese, 12 g), Arisaema erubescens (Dried radix of *Arisaema erubescens* (Wall.) Schott, Tiannanxing in Chinese, 6 g), Astragalus membranaceus (Dried radix of *Astragalus membranaceus* (Fisch.) Bunge., Huangqi in Chinese, 15 g), and Fructus aurantii (Dried fructus of Gleditsia sinensis lam (Dried spine of *Gleditsia sinensis Lam*, Zaojiaoci in Chinese, 10 g). The main components of the CFDT decoction were analyzed through quadrupole-time-of-flight mass spectrometry (UPLC-QQQ-MS) combined with high-performance liquid chromatography (Chromatographic column: Agilent ZORBAX SB-C18 column (100 × 2.1 mm, 1.9 μm); Injection quantity: 5 μL; Flow rate: 0.3 ml/min; Temperature: 20 ℃; Authenticated with standards). The process was guided by positive and negative ionization modes (Gas Temp: 325 ℃; Gas Flow: 7L/Min; Sheath Gas Temp: 350 ℃; Sheath Gas Flow: 11L/Min; Scan: 100–1500 Da; Fragment: 80–185 V; eV: 4–80 eV). The data were analyzed using SCIEX OS software. The compounds were identified according to the mass spectrometry data and matched to the TCM MS/MS Library.

### Experimental animals

40 female and 16 male Sprague-Dawley (SD) rats (age, 8 weeks; body weight, 180–220 g) were acquired (Liaoning Changsheng Biotechnology Co., Ltd.; production license: SCXK (Liao) 2015–0001) and housed in cages at 20 ± 3°C temperature and 45–65% humidity, with cycles of 12-h light/12-h dark (lights off, 18:00 h). All of the rats were given food and water ad libitum.

Animal processing procedures and the experimental design were authorized by the Ethical Committee of Animal Handling (2021-210, March 8, 2021) of Liaoning University of Traditional Chinese Medicine, Shenyang, China. We complied with the guidelines of Use and Care of Laboratory Animals published by the US National Institutes of Health and tried our best to decrease the number of rats and alleviate their suffering. At the same time, we tried to provide a

better environment for rats in this study.

40 female rats were randomly divided into four groups, namely, control group, PCOS group, PCOS+CFDT group, and PCOS+Metformin group. The rats in the control group were fed a normal-fat diet, intraperitoneally injected with 0.5% carboxymethyl cellulose (CMC, 1 mL/kg/d) for 21 days, and orally given saline (1 mL/kg/d) for the next 4 weeks. To build the PCOS rat model: the rats were fed a high-fat diet (HFD) and intraperitoneally injected with 1.0 mg/kg letrozole (1 mL/kg/d, dissolved in 0.5% CMC) for 21 days (13, 14). During this period, we recorded all rats’ body weights and estrous cycles. Vaginal smears were used to evaluate the onset of PCOS. The PCOS rats were further randomly divided into three groups, namely, PCOS group (intragastrically administered saline), PCOS+CFDT group (intragastrically administered the CFDT decoction, 15 g/kg/d), and PCOS+Metformin group (intragastrically administered metformin hydrochloride, 50 mg/kg/d) for 4 weeks. The dose of CFDT in our study is similar to that in Wang’ study (13). The constituents of the HFD included 13% fiber, 44% carbohydrates, 11% unsaturated fat, 25% total fat, and 18% protein, ash and other ingredients.

**Figure 1:**
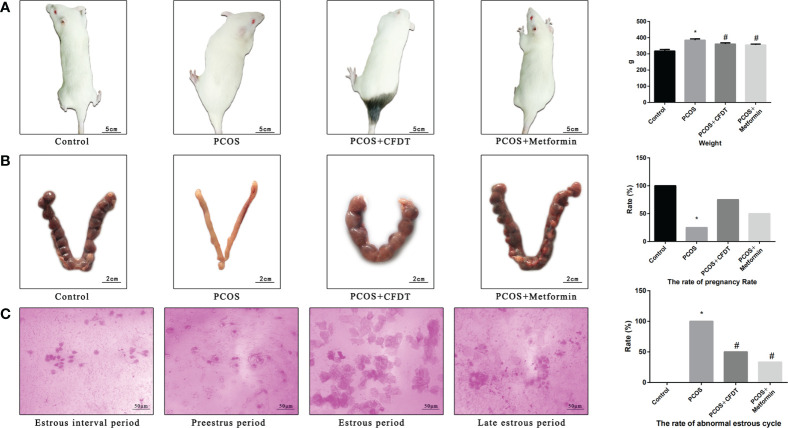
CFDT decoction improved the weight, rate of pregnancy, and the rate of abnormal estrous cycle in PCOS rats model. Rats were treated with CFDT decoction (15.0 g/kg.d) and metformin (50 mg/kg) followed by feeding with HFD and intraperitoneal injection with of letrozole (1 ml/kg/d) for 21 days. **(A)** Weight; **(B)** The rate of pregnancy (Female rats of each groups were sent to mate with male rats with a ratio of 1:1 for 12 h. The mixture of sperm and vaginal smears were seen on the next morning indicated the success of pregnancy, and this was considered as the 0.5th day of gestation. The pregnant rats were euthanized on the 13.5th day of the gestation) (n=4); **(C)** The rate of abnormal estrous cycle, estrous cycle of the rats (Estrous interval period: Vagina smear with white blood cells mainly; Preestrus period: With nuclear epithelial cells mainly; Estrous period: With Keratinized epithelial cells mainly; Late estrous period: See Keratinized epithelial cells and white blood cells) (n=6). Data are shown as mean ± SD. *p < 0.05 versus control group, ^#^ *p* < 0.05 versus PCOS group, ^△^ *p* < 0.05 versus PCOS+ CFDT group. δ The groups between the PCOS+ CFDT and PCOS+metformin do not have statistical difference.

Vaginal smears were analyzed every morning to observe the estrous cycle (Estrous interval period was mainly characterized by white blood cells; the proestrus period was mainly characterized by nuclear epithelial cells; the estrous period was mainly characterized by keratinized epithelial cells; the late estrous period was characterized by keratinized epithelial cells and white blood cells) (**Figure 1**). After the last irrigation, blood from the abdominal aorta of each group was collected on the non-estrus period with anesthesia (isoflurane *via* inhalation anesthesia) and oophorectomy. The rats were euthanized with the blood collected. Samples were permitted to coagulate for approximately 1 h at room temperature; then, serum was collected *via* centrifugation (2000 rpm/min) for 20 min and stored at -80°C for use. The ovarian tissues were fixed with 4% paraformaldehyde with the rest preserved at -80°C for expression analysis at genes and protein levels. The other four female rats of each group were mated with male rats at a ratio of 1:1 for 12 h (15). A mixture of sperm and vaginal smears on the following morning led to successful pregnancy, which was considered the 0.5th day of gestation. The pregnant rats were executed on the 15.5th day of gestation (15).

### Blood glucose detection and IR calculation

After fasting for 12 h, the rats were weighed. Tail vein blood was collected to measure fasting insulin (FINS) and fasting blood glucose (FBG) levels. HOMA-IR was formulated as IR = FBG

(mmol/L) × FINS (mU/L)/22.5.

The rats were euthanized. Then, blood from the abdominal aorta was collected. To obtain serum, blood was centrifuged at 3000 rpm (15 min) and then stored at –80°C. The ovaries were harvested, weighed, and then measured with a calliper.

### Ovarian index

The rats were euthanized. The ovaries were harvested and placed in a prechilled medium containing 1 mM EDTA, 10 mM Tris-HCl, and 250 mM sucrose, with pH of 7.4. Fatty tissues around the ovaries were removed. Then, the ovaries were weighed. The ovarian index was calculated as the ratio between the wet weight of bilateral ovaries (mg) and body weight (g) (16).

### Histological assessment of ovarian tissues

The ovarian tissues were fixed with paraformaldehyde (4%) for 24 h, dehydrated with different concentrations of ethanol, and then embedded in paraffin. Ovarian tissue blocks were cut to generate sections (5-μm-thick) (17). The cross-sections were stained in hematoxylin-eosin and then viewed with a light microscope (Olympus, CX33, Japan). All follicular phases (primary follicles, secondary follicles, and atretic follicles) and the corpora lutea were determined.

### Enzyme-linked immunosorbent assays

Arterial blood samples were used in enzyme-linked immunosorbent assays (ELISA) to measure follicle stimulating hormone [FSH, Sangon Biotech Co., Ltd. (D731057-0096)], luteinizing hormone [LH, Sangon Biotech Co., Ltd. (D731015-0096)], estradiol [E_2_, Biotech Co., Ltd. (EK7003)], testosterone [T, Biotech Co., Ltd. (EK7014)], interleukin-1β [IL-1β, Solarbio Life Sciences Co., Ltd. (SEKR-0002)], tumor necrosis factors-α [TNF-α, Solarbio Life Sciences Co., Ltd. (SEKR-0009)], C-reactive protein [CRP, Solarbio Life Sciences Co., Ltd. (SEKR-0017)], and interleukin-6 [IL-6, Solarbio Life Sciences Co., Ltd. (SEKR-0005)].

### Electron microscopy to observe mitochondria and cell apoptosis

Bilateral ovaries were harvested, minced into 1 mm^3^ fragments, fixed in 4% glutaraldehyde at 4°C for 2 h, and washed in 0.1 M sodium dimethylarsenate three times. The samples were postfixed with osmium tetroxide (1%) at 4°C for 90 min and washed with distilled water for three times, followed by dehydration in ethanol and acetone series. Finally, the samples were dehydrated in 100% propionaldehyde for two times. The samples were soaked in embedding agents at 1:3, 1:1, and 3:1. The samples were polymerized at 35°C (24 h), 45°C (24 h), and 60°C (24 h). The samples were trimmed with an ultrathin slicer (70–90 nm) to create a smooth surface. Double staining was carried out with lead citrate and uranyl acetate. The sections were observed using an electron microscope (H-7650; Hitachi, Tokyo, Japan).

### Apoptosis assay

The terminal deoxynucleotidyl transferase-mediated dUTP nick end-labeling (TUNEL) assay was used to assess apoptosis. For TUNEL staining, 5-μm-thick sections were employed. After deparaffinization and rehydration, the sections were treated with proteinase K (10 μg/mL) for 15 min and incubated with a reaction mixture at 37°C for 60 min in the dark. Following washing, the nuclei were stained with 4’,6-diamidino-2-phenylindole. The sections were observed with a fluorescence microscope (Canon, Japan). Eight random visual fields per sample were observed in a blinded manner to assess the number of TUNEL-positive cells.

### Caspase-3/9 activity

The AcDEVD-7-pNA substrate (Solarbio Co., Ltd.) was used to detect the caspase-9/3 activity. A 10 mg fragment of ovarian tissue was combined with the reaction buffer and then was incubated at 37°C for 2 h. A fluorimeter was used to quantify the enzyme-catalyzed release at a wavelength of 405 nm.

### Preparation of mitochondria suspension and detection of mitochondrial function

The rats were euthanized. Ovaries were harvested, placed in a prechilled medium containing Tris-HCl (10 mM), sucrose (250 mM), and EDTA (1 mM), with pH of 7.4, and homogenized on ice. The samples were consecutively centrifuged at 700 × g (10 min) and 7000 × g (10 min). Then, the mitochondria pellets were resuspended in 5 mL buffer mentioned above and then centrifuged (7000 × g) for 10 min twice. The highly pure mitochondrial pellets were resuspended in sucrose (20 mM), KH_2_PO_4_ (10 mM), MgCl_2_ (2 mM), KCl (100 mM), 5 mM HEPES (5 mM), and EDTA (1 mM). The BCA Protein Assay Kit was employed to measure the concentration of protein with the concentration of protein adjusted to 100–1000 μg/mL. The mitochondrial suspensions were used for measuring the MMP (18), opening of mitochondrial permeability transition pore (mPTP) (19), generation of ROS, degree of damaged mtDNA (20), mitochondrial oxygen consumption rate (21), respiratory control rate (RCR) (21), mitochondrial respiratory chain complex enzyme (I, II, III, IV, and V) levels (22), and the adenosine triphosphate (ATP) level (22).

### RNA extraction, cDNA dynthesis, and real-time qPCR

TRIzol reagent (Invitrogen, Carlsbad, CA, USA) was employed to separate the total RNA. A spectrophotometer was used to assess the RNA quality at a wavelength of 260 nm. The RNA was reverse-transcribed to cDNA using the M-MLV Reverse Transcriptase Kit (Promega, Madison, WI, USA) following to the manufacturer’s instructions. After that, real-time qPCR was performed on a Rotor-Gene Q Sequence Detection System (QIAGEN, Germany) using SYBR Premix Ex Taq II (TakaraBio) in line with the manufacturer’s instructions (23). The PCR requirements were as follows: 95°C (10 min) and 95°C (10 s), followed by 40 cycles at 60°C (15 s); 72°C (20 s); and 72°C (10 min). The relative mRNA expression of each gene was calculated using the GAPDH (24). **Table 1** listed the primer sequences.

**Table 1:** Sequence of primers for RT-PCR and long PCR.

Target Gene	Primer Sequence	Size (bp)	Tm (°C)
OPA1	Forward: 5’-TGGTTCGAGAGTCGGTTGAA-3’	189	56
	Reverse: 5’- CCTCCCAGTGCTTTGGAGTA -3’		56
Mfn1	Forward: 5’-GGGAAGACCAAATCGACAGA-3’	152	57
	Reverse: 5’-CAAAACAGACAGGCGACAAA-3’		57
Mfn2	Forward: 5’-GAGAGGCGATTTGAGGAGTG-3’	165	58
	Reverse: 5’-CTCTTCCCGCATTTCAAGAC-3’		56
Drp1	Forward: 5’-GCCCGTGGATGATAAAAGTG-3’	215	56
	Reverse: 5’-TGGCGGTCAAGATGTCAATA-3’		56
Fis1	Forward: 5’-AGATGGACTGGTAGGCATGG-3’	84	56
	Reverse: 5’-GACACAGCCAGTCCAATGAG-3’		56
PGC-1 α	Forward: 5’-GGACGAATACCGCAGAGAGT-3’	201	59
	Reverse: 5’-CCATCATCCCGCAGATTTAC-3’		56
Tfam	Forward: 5’-TCACCTCAAGGGAAATTGAAG-3’	241	55
	Reverse: 5’-CCCAATCCCAATGACAACTC-3’		56
Long Fragment	Forward:5’-AAAATCCCCGCAAACAATGACCACCC-3’	13400	72
	Reverse: 5’-GGCAATTAAGAGTGGGATGGAGCCAA-3’		72
Shrot Fragment	Forward: 5’-CCTCCCATTCATTATCGCCGCCCTGC-3’	235	60
	Reverse: 5’-GTCTGGGTCTCCTAGTAGGTCTGGGAA-3’		60
Bax	Forward: 5’-GCGATGAACTGGACAACAAC-3’	200	57
	Reverse: 5’-GATCAGCTCGGGCACTTTAG-3’		58
Bcl-2	Forward: 5’-CGAGTGGGATACTGGAGATGA-3’	236	58
	Reverse: 5’- GACGGTAGCGACGAGAGAAG-3’		59
Caspase-3	Forward: 5’-CCCATCACAATCTCACGGTAT-3’	195	57
	Reverse: 5’-GGACGGAAACAGAACGAACA-3’		58
Caspase-9	Forward: 5’-GCCTCTGCTTTGTCATGGAG-3’	181	56
	Reverse: 5’-AGCATGAGGTTCTCCAGCTT-3’		56
ASK1	Forward: 5’-ACAATGAGCAGACGATTGGC-3’	168	56
	Reverse: 5’-CAGCAAGCCTCTTGGATGTC-3’		56
JNK	Forward: 5’-TGGATTTGGAGGAGCGAACT-3’	69	56
	Reverse: 5’-TCACTGCTGCACCTAAAGGA-3’		56
Cyc-c	Forward: 5’-GGACAGCCCCGATTTAAGTA-3’	121	57
	Forward: 5’-TCAATAGGTTTGAGGCGACAC-3’		58
GAPDH	Forward: 5’- AGGTCGGTGTGAACGGATTTG -3’	20	58
	Reverse: 5’- GGGGTCGTTGATGGCAACA-3’		58

### Protein detection

RIPA Buffer was used to extract total proteins from ovaries. The BCA Protein Assay Kit was employed to detect the concentration of protein. Equivalent amounts of total protein were subjected to 8–12% sodium dodecyl sulfate-polyacrylamide gel electrophoresis. The proteins were transferred to PVDF membranes. After blocking with skim milk, the membranes were incubated overnight with an anti-GAPDH, anti-ASK1, anti-p-ASK1, anti-JNK, anti-p-JNK, anti-Bcl-2, anti-Bax, anti-caspase-9/3, anti-Cyt-c, anti-OPA1, anti-Mfn1, anti-Mfn2, anti-Drp1, anti-Fis1, or anti-PGC1a antibody (**Table 2**). After that, the membranes were incubated with a secondary HRP-conjugated goat anti-rabbit antibody (Santa Cruz Biotechnology, Santa Cruz, CA, USA). The Enhanced Chemiluminescence Kit (Thermo Fisher Scientific) was used to visualize the proteins. Alpha View Software (Cell Biosciences, Preston VIC, Australia) was used for densitometric analysis.

**Table 2:** Antibodies used in the study.

Antibodies	Manufacturer	Catalogue No.	Observed MW	Dilution
Anti-ASK1	Proteintech	67072-1-1g	110 KDa	1:2000
Anti-p-ASK1	Proteintech	28846-1-AP	120 KDa	1:1000
Anti-JNK	Proteintech	10176-2-AP	46KDa	1:2000
Anti-p-JNK	Proteintech	80024-1-RR	46 KDa	1:2000
Anti-Bcl-2	Proteintech	26593-1-AP	26 KDa	1:1000
Anti-Bax	Proteintech	50599-2-1g	26 KDa	1:6000
Anti-Caspase-3	Proteintech	19677-1-AP	32 KDa	1:1000
Anti-Caspase-9	Proteintech	10380-1-AP	47 KDa	1: 500
Anti-Cyt-c	Proteintech	12245-1-AP	13 KDa	1:3000
Anti-OPA1	Proteintech	66583-1-Ig	100 KDa	1:1000
Anti-Mfn1	Proteintech	13798-1-AP	86 KDa	1:500
Anti-Mfn2	Proteintech	12186-1-AP	86 KDa	1:3000
Anti-Drp1	Proteintech	10656-1-AP	27 KDa	1:1000
Anti-Fis1	Proteintech	66635-1-Ig	15 KDa	1:3000
Anti-PGC1a	Proteintech	66369-1-1g	100 KDa	1:5000
Anti-GAPDH	Proteintech	60004-1-1g	36 KDa	1:20000

### Statistical analysis

Statistical analysis was performed using SPSS 17.0 Software (SPSS Inc. Chicago, IL, USA). The data were expressed as mean ± standard deviation. One-way analysis of variance (ANOVA) was used to compare four independent groups. Two-to-two comparison among groups was employed to analyze the variance. The LSD-t test was employed to compare multiple comparisons among four groups. We defined *p* < 0.05 as having a statistically significant difference.

## Results

### Chemical components of the CFDT decoction

The chemical components of the CFDT decoction were measured *via* UPLC-QQQ-MS. They were as follows: narirutin (content: 24.7403 μg/g; CAS: 14259-46-2), chrysin-7beta-monoglucoside (content: 0.74 μg/g; CAS: 31025-53-3), liquiritin (content: 99.97 μg/g; CAS: 551-15-5), nobiletin (content: 39.93 μg/g; CAS: 478-01-3), hesperetin (content: 96.28 μg/g; CAS: 520-33-2), tanshinone IIA (content: 4.92 μg/g; CAS: 568-72-9), calycosin (content: 1.30 μg/g; CAS: 20575-57-9), formononetin (content: 1.83 μg/g; CAS: 485-72-3), stachydrine (content: 4.36 μg/g; CAS: 471-87-4), betaine (content: 61.97 μg/g; CAS: 107-43-7), astragaloside IV (content: 80.79 μg/g; CAS: 84687-43-4), acteoside (content: 26.25 μg/g; CAS: 61276-17-3), rosmarinic acid (content: 6.00 μg/g; CAS: 20283-92-5), and rhein (content: 1.64 μg/g; CAS: 478-43-3). Standard curve of compounds were showed in **Table 3**.

**Table 3:** Standard curve of compounds.

Compounds	LLOQ (μg·mL-1)	linear ranger (μg·mL-1)	Linear regression equation	Correlation coefficient (r)
Narirutin	0.115	0.1148-58.8	Y=0.078X+0.0027	0.9998
Calycosin-7-O-β-D-glucoside	0.011	0.011-14.71	Y=6.1821X-0.7872	0.9990
Liquiritin	0.011	0.011-7.35	Y=0.4695X- 0.0299	0.9995
Nobiletin	0.011	0.011-3.68	Y=106.77X-5.8926	0.9970
Hesperetin	0.011	0.011-7.35	Y=0.3739X-0.1658	0.9988
Tanshinone IIA	0.011	0.011-58.8	Y=0.228X-0.0075	0.9989
Calycosin	0.011	0.011-3.68	Y=7.4149X-0.0274	0.9979
Formononetin	0.011	0.011-0.92	Y=3.3123X-0.0241	0.9997
Stachydrine	0.011	0.011-7.35	Y=0.1754X+0.0506	0.9998
Betaine	0.114	0.114-58.8	Y=0.2471X+1.0458	0.9983
Dioscin	0.011	0.011-58.8	Y=0.0088X+0.0114	0.9996
Astragaloside IV	0.115	0.115-58.8	Y=0.0003X+0.0006	0.9951
Acteoside	0.92	0.92-58.8	Y=0.0051X-0.0027	0.9980
Rosmarinic acid	0.011	0.0114-58.8	Y=0.0266X-0.0211	0.9990
Rhein	0.011	0.0114-58.8	Y=0.036X+0.0319	0.9988

### The CFDT decoction improved the weight, rate of pregnancy, and the rate of the abnormal estrous cycle in the PCOS rat model

To assess the severity of PCOS and the protective effects of the CFDT decoction, we examined the body weight (**Figure 1**), rate of pregnancy (**Figure 1**), and rate of the abnormal estrous cycle (**Figure 1**). The body weight increased in PCOS group compared to control group (*p* < 0.05), but decreased after administration of the CFDT decoction and metformin (*p* < 0.05) (**Figure 1**). The rate of pregnancy decreased in the PCOS group compared to the control group (*p* < 0.05) but increased after administration of the CFDT decoction and metformin (*p* < 0.05) (**Figure 1**). Compared to the control group, the rate of abnormal estrous cycles increased in the PCOS group (*p* < 0.05), but decreased after administration of the CFDT decoction and metformin (*p* < 0.05). Daily vaginal smears displayed that the rats in the control group had regular estrous cycles (4–5 days) that included proestrus, estrus, metestrus, and diestrus phases, whereas the rats in the PCOS group showed irregular estrous cycles, in diestrus period, showing predominantly leukocytes (**Figure 1**).

### The CFDT decoction improved the ovarian index, ovarian diameter, follicular phases, serum hormones, blood glucose indices, and inflammatory cytokine levels in the PCOS rat model

**Figure 2:**
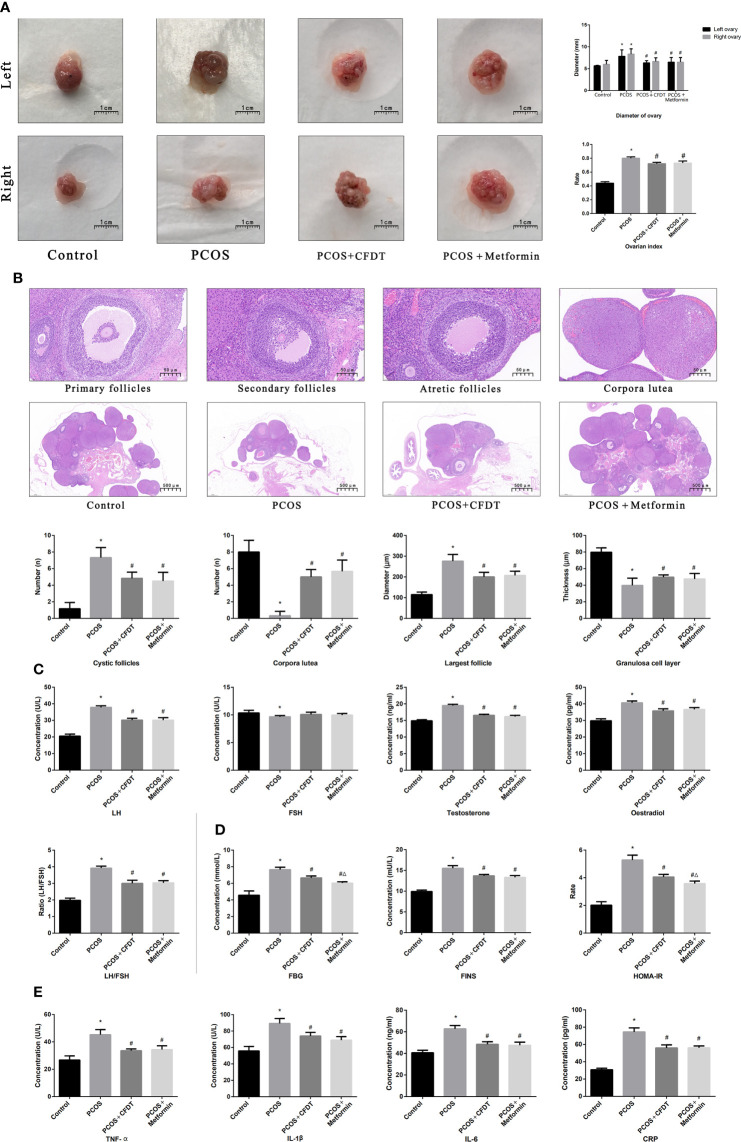
CFDT decoction improved the ovarian index, ovarian diameter, the number of all phases of follicles, serum hormones, blood glucose indices, and inflammatory factors in PCOS rats model. Rats were treated with CFDT decoction (15.0 g/kg.d) and metformin (50 mg/kg) followed by feeding with HFD and intraperitoneal injection with of letrozole (1 ml/kg/d) for 21 days. **(A)** Ovarian index (Ovarian index= wet weight of bilateral ovaries (mg)/body weight (g)×100%, the size of the ovary in PCOS group was significantly reduced compared with the control group) and ovarian diameter, the scale bars represent a length of 1 cm on histology; **(B)** Histological assessment of the ovarian tissue using hematoxylin-eosin (HE) staining [All phases of follicles (primary follicles, secondary follicles, and atretic follicles) and corpora lutea were counted], the scale bars represents a length of 20 μm on histology; **(C)** Serum hormones (LH, FSH, T, and E_2_); **(D)** Blood glucose indices (FBG, FINS, and HOMA-IR); **(E)** Serum inflammatory factors (TNF- α, IL-1β, IL-6, and CRP). Data are shown as mean ± SD. *p < 0.05 versus control group, ^#^ *p* < 0.05 versus PCOS group, ^△^ *p* < 0.05 versus PCOS+ CFDT group. (n=6).

We examined the ovarian diameter and index (**Figure 2**), follicular phases (**Figure 2**), serum hormone levels (**Figure 2**), blood glucose indices (**Figure 2**), and inflammatory cytokine levels (**Figure 2**). The ovarian diameter and index increased in the PCOS group compared to control group (*p* < 0.05), but decreased after administration of the CFDT decoction and metformin (*p* < 0.05) (**Figure 2**). Light microscopy results showed the primary, secondary, and atretic follicles, the corpora lutea, and the normal granulosa cells in the ovaries of control rats. By contrast, early-stage and atretic follicles were observed in rats with PCOS, in addition to many large cysts and few granulosa cells. The corpora lutea and granulosa cells were visible after the administration of the CFDT decoction and metformin (**Figure 2**). Serum LH, T, and E_2_ levels increased in the PCOS group compared to the control group (*p* < 0.05), but decreased after the administration of the CFDT decoction and metformin (*p* < 0.05). No difference in the FSH level was detected among the four groups (**Figure 2**) (*p* > 0.05). FBG, FINS, and HOMA-IR increased in PCOS group (*p* < 0.05), but decreased after the administration of the CFDT decoction and metformin (*p* < 0.05), and metformin was more effective than the CFDT decoction (*p* < 0.05) (**Figure 2**). TNF- α, IL-1β, IL-6, and CRP levels increased in the PCOS group (*p* < 0.05), but decreased after the administration of the CFDT decoction and metformin (*p* < 0.05) (**Figure 2**).

### The CFDT decoction improved mitochondrial function in the PCOS rat model

**Figure 3:**
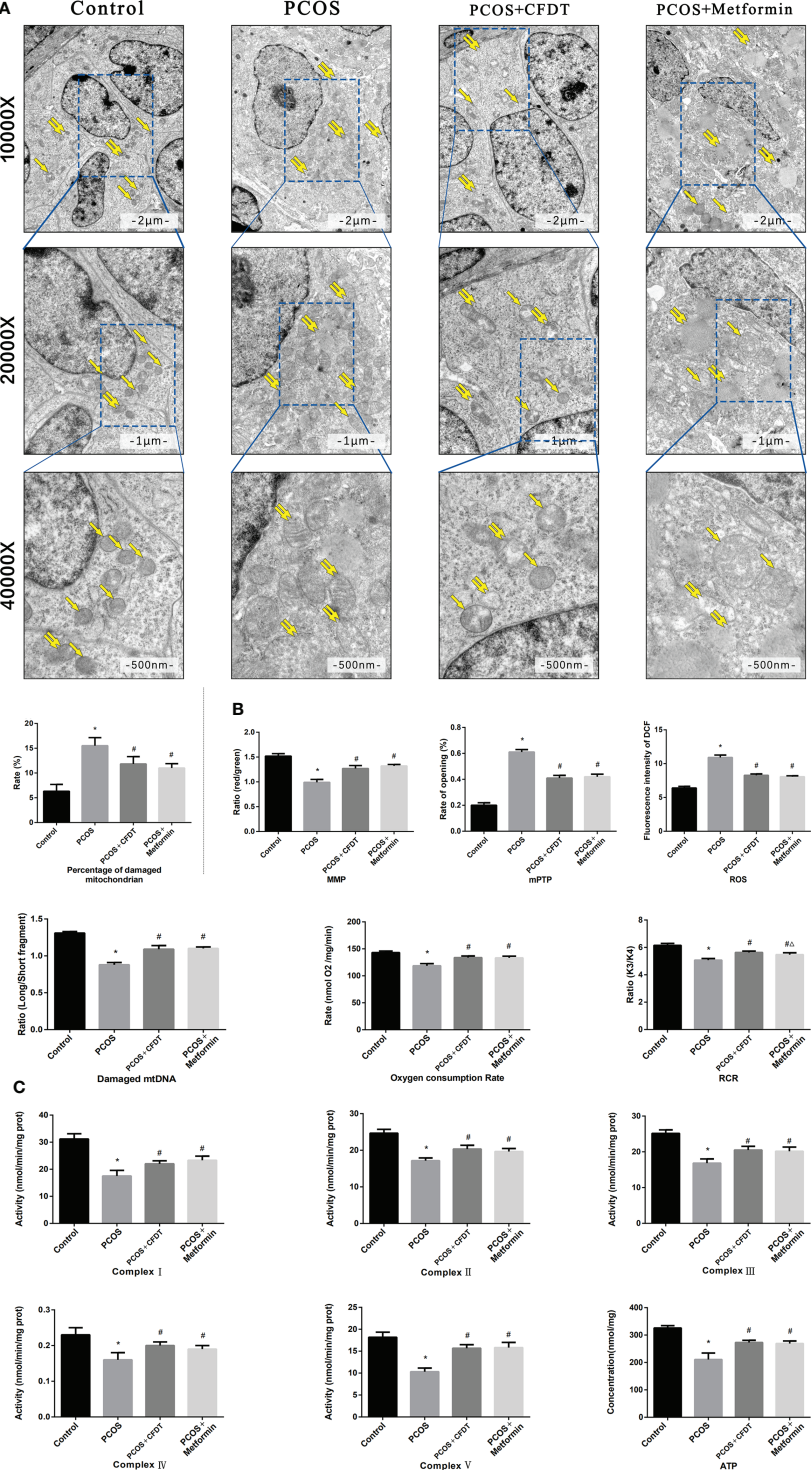
CFDT decoction improved mitochondrial function in PCOS rats model. Rats were treated with CFDT decoction (15.0 g/kg.d) and metformin (50 mg/kg) followed by feeding with HFD and intraperitoneal injection with of letrozole (1 ml/kg/d) for 21 days. **(A)** Electron microscope pictures (10,000×; 20,000×; 40 000×) of the ovary in PCOS rats, the scale bars represents a length of 2 μm, 1 μm, and 500 nm on histology respectively. Abnormal mitochondrial (paired yellow arrow) morphology showed mitochondrial membrane rupture or swellings, and normal mitochondrial (single yellow arrow) morphology type showed smooth mitochondrial membrane and distinct inner carinulae and percentage of damaged mitochondria; **(B)** The MMP (ratio of red/green), the opening of mPTP (%), the mitochondrial ROS, the mtDNA damage (ratio of long/short fragments), the mitochondrial RCR, mitochondrial oxygen consumption rate; **(C)** The mitochondrial respiratory chain complex enzymes (I, II, III, IV, and V) and ATP were recorded above. Data are shown as mean ± SD. *p < 0.05 versus control group, ^#^ *p* < 0.05 versus PCOS group, ^△^ *p* < 0.05 versus PCOS+ CFDT group. (n=6).

The mitochondrial function was assessed by electron microscopy. The results showed intact mitochondria but damaged mitochondria, with membrane swelling and rupture, in the PCOS group. The percentage of damaged mitochondria of ovaries was higher in the PCOS group compared to the control group (*p* < 0.05); however, it decreased after the administration of the CFDT decoction and metformin (**Figure 3**).

We measured the MMP (ratio of red/green), the opening of mPTP (%), ROS production, degree of mtDNA damage, oxygen consumption rate, RCR, the ATP level, and the activity of mitochondrial respiratory chain complex enzymes (I, II, III, IV, and V) to further evaluate the mitochondrial function of ovaries. ROS production and the opening of mPTP (%) increasedin the PCOS group compared to the control group (*p* < 0.05). Again, both parameters decreased after the administration of the CFDT decoction and metformin (*p* < 0.05). MMP (ratio of red/green), mitochondrial oxygen consumption rate, and RCR decreased in the PCOS group compared to the control group (*p* < 0.05), but increased after administration of CFDT decoction and metformin (*p* < 0.05). The ratio of long-to-short fragments of mtDNA was measured by Real-time qPCR. The ratio of long/short fragments decreased in the PCOS group compared to the control group (*p* < 0.05). However, administration of CFDT decoction and metformin increased the ratio (*p* < 0.05). The activity of mitochondrial respiratory chain complex enzymes (I, II, III, IV, and V) and the ATP level decreased in the PCOS group compared to the control group (*p* < 0.05). However, administration of CFDT decoction and metformin increased these indices (*p* < 0.05) (**Figure 3B**).

### The CFDT decoction affected the mitochondrial function of ovarian in the PCOS rat model

**Figure 4:**
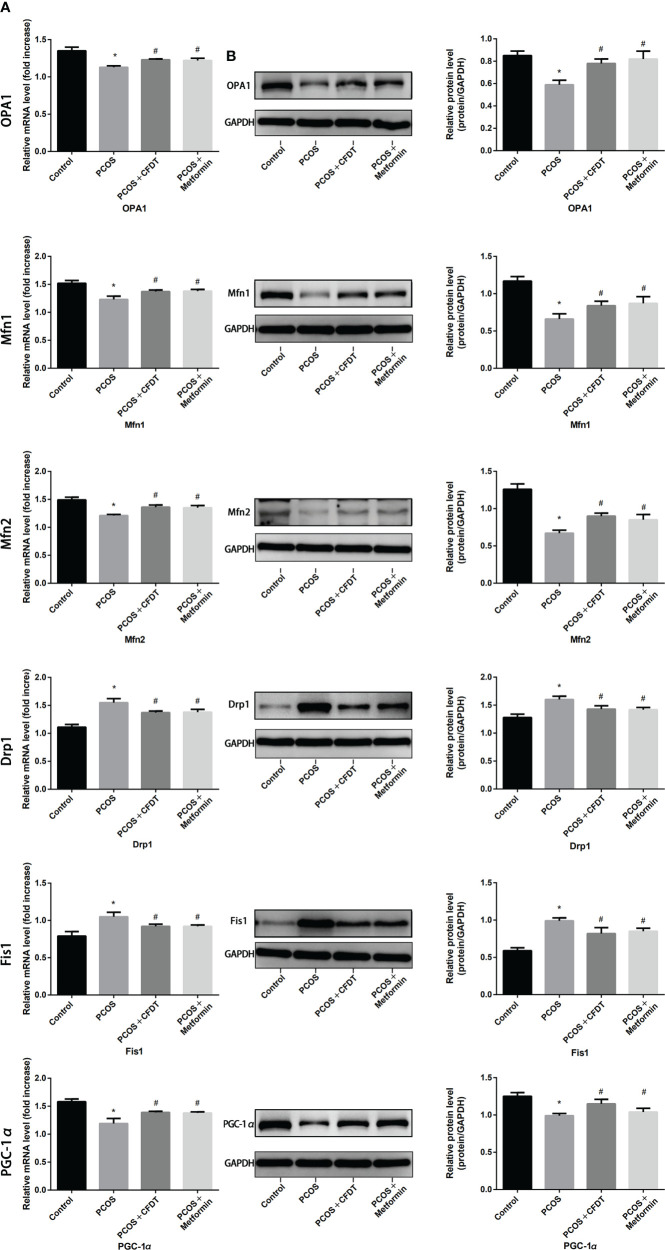
CFDT decoction improved mitochondrial biogenesis and dynamics in PCOS rats model. Rats were treated with CFDT decoction (15.0 g/kg.d) and metformin (50 mg/kg) followed by feeding with HFD and intraperitoneal injection with of letrozole (1 ml/kg/d) for 21 days. We used real-time qPCR and western blot to detect mitochondrial biogenesis and dynamics. We chose OPA1, Mfn1, and Mfn2 to represent mitochondrial biogenesis function, PGC-1 α to represent the dynamic mitochondrial fusion, and Drp1 and Fis1 to represent mitochondrial fission. The expression of OPA1, Mfn1, Mfn2, PGC-1 α, Drp1, and Fis1 at mRNA **(A)** and protein **(B)** levels. Data are shown as mean ± SD. *p < 0.05 versus control group, ^#^ *p* < 0.05 versus PCOS group, ^△^ *p* < 0.05 versus PCOS+ CFDT group. (n=6).

The genes expression was examined by qPCR (mRNA) and Western blotting (protein). OPA1, Mfn1, and Mfn2 were selected as markers of mitochondrial biogenesis, with PGC-1 α as a marker of mitochondrial fusion and Drp1 and Fis1 as markers of mitochondrial fission, respectively. OPA1, Mfn1, Mfn2, and PGC-1 α expression decreased in the PCOS group compared to the control group (*p* < 0.05), but increased after administration of CFDT decoction and metformin (*p* < 0.05). Drp1 and Fis1 expression increased in PCOS group compared to the control group (*p* < 0.05), but decreased after the administration of the CFDT decoction and metformin (*p* < 0.05) (**Figures 4A, B**).

### CFDT decoction modulated the ASK1/JNK pathway in the PCOS rat model

**Figure 5:**
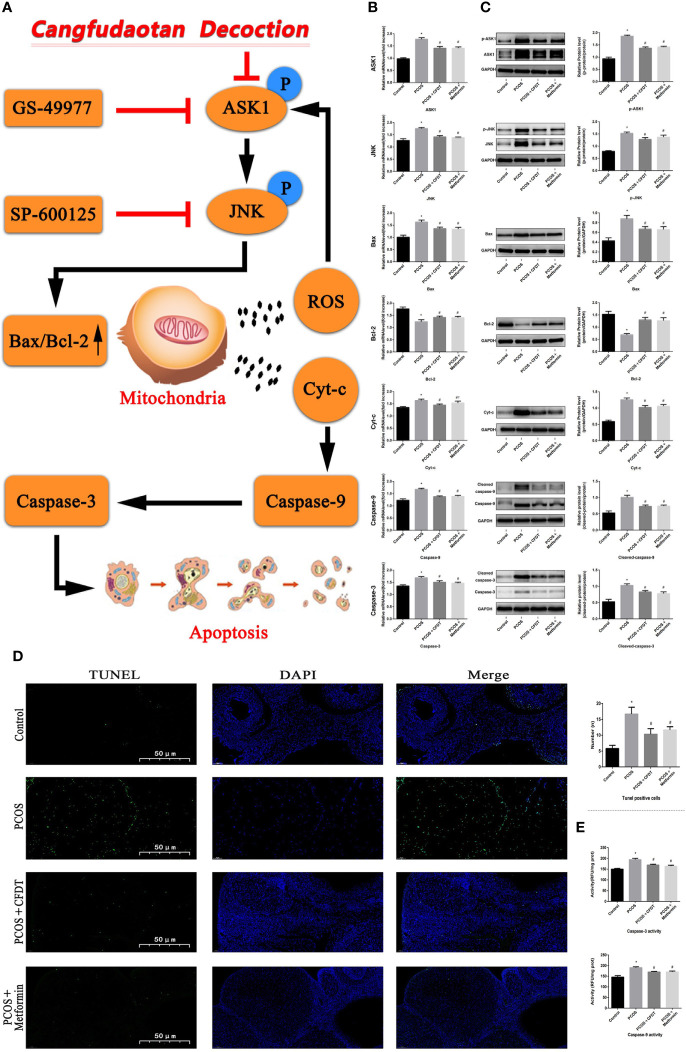
CFDT decoction improved mitochondrial function and inhibited apoptosis *via* inhibiting the ASK1/JNK pathway. Rats were treated with CFDT decoction (15.0 g/kg.d) and metformin (50 mg/kg) followed by feeding with HFD and intraperitoneal injection with of letrozole (1 ml/kg/d) for 21 days. **(A)** Graphical abstract: Mitochondrial dysfunction of the ovarian granular cells occurs following PCOS and can lead to granular cells apoptosis. CFDT decoction can relieve granular cell apoptosis by improving mitochondrial function *via* inhibiting the ASK1/JNK pathway *in vivo*. We suggested that CFDT decoction be used as a potential therapeutic agent for PCOS and mitochondria are seen as a potential therapeutic target. We used real-time qPCR and western blot to detect the target genes of the ASK1/JNK pathway at mRNA **(B)** and protein **(C)** levels. **(D)** TUNEL positive cells, the scale bars represent a length of 50 μm on histology; **(E)** The activity of caspase-9/3. Data are shown as mean ± SD. *p < 0.05 versus control group, ^#^ *p* < 0.05 versus PCOS group, ^△^ *p* < 0.05 versus PCOS+ CFDT group. (n=6).

The levels of target genes in the ASK1/JNK pathway were examined by qPCR (mRNA) and Western blotting (protein). Caspase-9/3, Bax, ASK1, JNK, and Cty-C mRNA expression increased in the PCOS group compared to the control group (*p* < 0.05), whereas the mRNA level expression of these indices decreased after treatment with the CFDT decoction and metformin (*p* < 0.05). PCOS reduced the mRNA level expression of Bcl-2 compared with the control group (*p* < 0.05). The mRNA level expression of Bcl-2 increased after administration of CFDT decoction and metformin (*p* < 0.05). CFDT decoction and metformin inhibited ASK1 and JNK phosphorylation (decreased p-ASK1/ASK1 and p-JNK/JNK) (**Figure 5B**). The number of apoptotic granulosa cells in the PCOS group increased compared to the control group (*p* < 0.05). However, treatment with the CFDT decoction and metformin decreased the number of apoptotic cells (**Figure 5D**). Caspase-9/3 activity in ovaries of the PCOS group was higher than that of the control group (*p* < 0.05). However, administration of CFDT decoction and metformin decreased the activity of caspase-9/3 (**Figure 5**).

## Discussion

Our main finding is that CFDT decoction can reduce granulosa cell apoptosis in the PCOS rat model. We also defined the potential protective effects and the mechanism of action of CFDT decoction. Mitochondrial dysfunction may induce granulosa cell apoptosis, whereas the CFDT decoction can reduce apoptosis by improving mitochondrial function *via* inhibition of the ASK1/JNK pathway. To the best of our knowledge, this is the first study to investigate the treatment mechanism of CFDT decoction. To examine the protective mechanism of CFDT decoction, we used HFD and letrozole to establish a PCOS rat model.

The incidence of PCOS continues to increase in females at reproductive age, but the pathogenesis of PCOS remains complex and unclear. There is increasing evidence that IR is related to the pathogenesis of PCOS (25–27). In this study, in addition to the obviously increased HOMA-IR in PCOS rat model, the serum T level, and LH/FSH ratio, significantly increased, which is consistent with results from an earlier study (27). The increase in insulin level can up-regulate androgen levels, thereby inducing hyperandrogenism and up-regulating androgen levels in fatty tissues of PCOS patients *via* increased AKR1C3 activity (26). An irregular estrous cycle (prolonged diestrus period) of the PCOS group was detected based on vaginal smears. Histological analysis showed that the morphology of ovaries was characterized by fewer granulosa cells and oocytes and cystic dilatation of the follicles in the PCOS group, compared with the control group. Hyperandrogenism can induce early luteinization of granulosa cells, inhibit follicular development and growth, and trigger follicle atresia, thereby leading to poor ovulation or anovulation, which is all consistent with the earlier study (28). However, we observed an increased E_2_ level in the PCOS rat model. The reason for this phenomenon may be that PCOS causes ovarian injury such that the negative feedback regulates the pituitary gland to increase the secretion of E_2_. In addition, we believe that the created PCOS rat model reflects the early stage of PCOS, while clinical patients often suffer from the disease for a long time, where negative feedback regulation is weakened.

After the administration of the CFDT decoction, FPG and HOMA-IR decreased, indicating that the CFDT decoction improved IR in the PCOS rat model. In the PCOS+ CFDT group, serum LH and T levels decreased and the damage of ovarian morphology was alleviated. The effect was similar to that of metformin, which is consistent with the earlier study (27).

As a low-grade chronic inflammatory disease, PCOS often associated with permanently elevated levels of inflammatory markers (TNF- α, CRP, IL-6, IL-8, and IL-18) in patients, and inflammatory markers can induce mitochondrial dysfunction (29, 30). Multiple inflammatory factors, such as CRP, IL-6, TNF- α, IL-8, IL-1, MCP, and CCL_2_, increase OS, and inflammatory immune cell infiltration can lead to a low-grade inflammatory response (31). Thus, relieving inflammation may be necessary for treating PCOS. In this study, we showed that the serum levels of IL-1, TNF- α, CRP, and IL-6 increased in the PCOS group, but decreased after the CFDT decoction and metformin administration.

The granulosa cells bring growth factors and hormones, interact with oocytes, build a highly specialized microenvironment, and perform essential roles in follicle development and maturation (32). The hallmark of PCOS is follicular dysplasia, which has been reported in various studies to be associated with granulosa cell apoptosis (33–35). In the PCOS group, the ovaries demonstrated atretic follicles and small follicles at an early stage of development. In addition, there were many large cysts with few granulosa cells. These morphological changes were reversed by administering the CFDT decoction or metformin.

There is no specific Chinese medical term “PCOS” in Chinese medicine books. According to the clinical characteristics, PCOS belongs to the categories of infertility, irregular menstruation, and obesity. TCM has been used to treat menstrual disorders, infertility, and obesity (36, 37). A previous study showed that herbal medicines could alleviate some symptoms of PCOS (38). Analysis of network pharmacology, which focused on seeking the related pathways and chemical compositions of the CFDT decoction for treating PCOS, showed that IR, as well as PI3K-Akt, MAPK, HIF-1 signaling pathways, and Toll-like receptor, was involved in the treatment of PCOS (39). The CFDT decoction is a herbal medicine that has extensive clinical application prospects for PCOS patients (12, 40). Chemical profiling analysis and quality control of the CFDT decoction have been performed (27).

The production of ROS and release of Cyt-c can induce apoptosis, while the opening of mPTP is key to the production of ROS and release of Cyt-c from mitochondria to the cytoplasm; thus, inhibiting the mPTP opening can improve the mitochondrial function and decrease apoptosis (41). It is believed that mitochondrial dysfunction (especially mPTP opening) is targeted in treating ovarian cancer (42). The opening of mPTP induces a decreased MMP, mitochondrial swelling, and inhibition of oxidative phosphorylation. The opening of mPTP operates *via* binding to CyP-D on the inner mitochondrial membrane. Cyclosporine A (CsA) restrains the mPTP opening *via* combining with CyP-D and decreasing injury (43). We hypothesize that the CFDT decoction might play a protective function in granulosa cells *via* increasing mitochondrial function (inhibiting the mPTP opening).

Apoptosis is an ATP-dependent cell death induced by a variety of extracellular and intracellular signals (26, 35, 44). The apoptosis of ovarian oocytes and granulosa cells can induce follicular atresia. A previous study has reported that follicular atresia is a feature of PCOS (45) and that granulosa cell apoptosis is an initiating factor of PCOS (46). Although many apoptotic pathways have been studied, the mitochondrial pathway is the most common apoptotic cascade (47). Previous study showed that apoptosis was increased in PCOS rats and that treatment with the CFDT decoction and metformin could reduce apoptosis, indicating that the CFDT decoction and metformin might ameliorate follicular development in rats with PCOS by modulating apoptosis (13). To further explore the anti-apoptotic mechanism of the CFDT decoction, TUNEL staining, caspase-9/3 activity, and apoptotic genes (Cyt-c, Bcl-2, and Bax) expression were assessed. Our results demonstrated that the number of apoptotic cells in ovaries in the PCOS group increased compared to the control group, and that treatment with the CFDT decoction and metformin could reduce apoptosis. The decreased Bax shifted to mitochondria, integrating with the increased Bcl-2 to form the Bax/Bcl-2 heterodimer on the mitochondrial membrane, and as such promoted the anti-apoptosis effect. As a result, the MMP was steady with little Cyt-c released into the cytoplasm. The Bcl-2 family contains pro-apoptosis molecules (Bad, Bax, and Bid) and anti-apoptosis genes (Bcl-xL, Bcl-2, and Bel-w) (48). Bcl-2, a proto-oncogene, can inhibit cancer cell apoptosis, while Bax is a pro-apoptotic gene. A previous study demonstrated that the Bcl-2/Bcl-2 homodimer and the Bax/Bcl-2 heterodimer could promote mitochondrial membrane permeability and that the Bax/Bax homodimer could decrease the MMP. The ratio of Bax/Bcl-2 may affect apoptosis (48).

The senescence and apoptosis of granulosa cells are responsible for the decline in ovarian reserve in PCOS (49). Granulosa cell injury is generally associated with mitochondrial dysfunction in PCOS (50). Mitochondrial apoptotic signaling pathways (NF-jB/p53/PUMA and PI3K/Akt/Bad) play an essential role in organ injury, which in turn can induce mitochondrial dysfunction (51–53). In this study, PCOS was associated with morphological changes in mitochondria (swelling or membrane rupture) and increase in the percentage of damaged mitochondria, but CFDT decoction was able to protect the mitochondria. An imbalance of mitochondrial homeostasis plays an essential function in the pathophysiology of PCOS (54). Furthermore, impaired ovarian mitochondrial function and increased OS are critical elements in PCOS (50).

In this study, we firstly established a PCOS rat model. Then, we used CFDT decoction to investigate the MMP, opening of mPTP (%), Cty-c release, ROS production, degree of mtDNA damage, oxygen consumption rate, ATP level, RCR, and activity of mitochondrial respiratory chain complex enzymes (I, II, III, IV, and V). RCR, MMP, oxygen consumption rate, mitochondrial respiratory chain complex enzymes (I, II, III, IV, and V), and ATP levels were reduced in the PCOS group. The ROS production and the opening of mPTP (%) increased in the PCOS group, whereas mitochondrial function indices were improved after treatment with the CFDT decoction. As the copy number of mtDNA in every mitochondrion was steady, the total copy number of mtDNA was applied to evaluate the number of mitochondria (20). Although the mechanism of restoring mtDNA is ambiguous, mtDNA is close to the respiratory chain complex enzymes, which, therefore, is more brittle when exposed to oxidative reactions. Furthermore, we detected damaged mtDNA by calculating the ratio of long/short fragments. The ratio of long/short fragments was reduced in the PCOS group, but increased after treatment with the CFDT decoction.

Mitochondrial biogenesis involves the function of many genes, such as OPA1, Mfn1, and Mfn2. As a regulatory gene, optic atrophy 1 (OPA1) plays an essential role in managing the mitochondrial dynamics and other related roles. In addition, the overexpression of L-OPA1 can reduce neuronal apoptosis *via* increasing the ratio of Bcl-2/Bax and decreasing the caspase-3 level. The overexpression of L-OPA1 can modulate mitochondrial dysfunction by reducing oxidative stress and mitochondrial bioenergetics deficits, preserving mitochondrial integrity, and promoting mitochondrial biogenesis in brain tissues (55). Mitochondrial function is also under the control of mitofusins (Mfns) and dynamin-related protein 1 (Drp1) (56). Mitofusin2 (Mfn2), a conserved dynamin-like GTPase situated in the outer membrane of mitochondria, affects mitochondrial structure and function by modulating fission and fusion (57). Furthermore, Mfn2 preserves cell function by modulating the respiratory chain, MMP, metabolic processes, and apoptosis (58). Mfn2 plays a crucial role in maintaining the integrity of mtDNA (59). As shown in this study, OPA1, Mfn1, Mfn2, mRNA and protein levels decreased in the PCOS group, but increased after treatment with the CFDT decoction. These results further corroborated previous research that Mfn2 can maintain the integrity of mtDNA (59). Mitochondria undergo fission and fusion, and these dynamic processes are essential for maintaining size and shape. PPARγ coactivator-1- α (PGC-1 α) is a crucial transcriptional co-activator that modulates critical factors containing Tfam and Nrf1 which facilitate mitochondrial biogenesis (60). Drp1, the significant regulation of mitochondrial fission, is a cytosolic member of the dynamin family of GTPases. Drp1 binds to mitochondrial membranes, and its level increases during apoptosis (61). FiS1 induces apoptosis *via* the interaction with endoplasmic reticulum-localized Bap31, leading to Cyt-c release and ultimately cell death (62). In addition, FIS1 has been implicated in reducing the GTPase activity of Mfn1, Mfn2, and OPA1 (63). Our results showed that the expression of Drp1, FiS1, and PGC-1 α changed in PCOS, showing a chaotic fission-fusion balance in mitochondria. We observed an increase in Drp1 and FiS1 levels and a decrease in the PGC-1 α level in PCOS. The CFDT decoction could decrease the PCOS-induced imbalance by increasing mitochondrial biogenesis and renewing the balance between fusion and fission.

Apoptosis signal-regulating kinase 1 (ASK1), a kind of universally expressed mitogen-activated kinase kinase kinase (MAP3K), can be activated by all kinds of stimuli, and then activate downstream kinases (JNK and p38) (64). As such, it can play an important role in neurodegenerative disorders, cancer, and inflammatory diseases (65). The ASK1 inhibitor selonsertib (GS-49977) is a possible treatment drug during the early treatment of ALF through reducing JNK-mediated Drp1 mitochondrial translocation and then remedying mitochondrial injury (66). The ASK1/JNK signalling pathway plays a crucial function in inducing mitochondrial-induced apoptosis (67). In this study, CFDT decoction affected the expression of ASK1/JNK pathway-related proteins, inhibited ASK1 and JNK phosphorylation, and downregulated Cyt-c expression and cleaved caspase-9/3, indicating that the CFDT decoction could improve mitochondrial function and inhibit apoptosis through the ASK1/JNK pathway.

## Conclusions

In this study, we used isolated mitochondria and showed that PCOS promoted injury of the mitochondria in the ovary and damaged mtDNA and mitochondrial function, including respiratory, biogenesis, and the ROS balance. The opening of mPTP caused mitochondrial swelling and induced flow back of protons from the mitochondrial membrane space to the matrix, thereby reducing ATP synthesis and the MMP and inducing metabolic abnormalities or even apoptosis. The CFDT decoction could inhibit apoptosis *via* improving mitochondrial function through inhibition of ASK1/JNK pathway in PCOS rats.

## Data availability statement

The datasets used and/or analyzed during the current study are available from the corresponding author on reasonable request.

## Ethics statement

The animal study was reviewed and approved by Ethical Committee of Animal Handling (2021-210, March 8 2021) of Liaoning University of Traditional Chinese Medicine.

## Author contributions

XLJ and HT wrote the manuscript and researched data. X-SX, S-BQ, S-YZ, and L-NZ selected rats and extracted blood. HT dealed with the figures. S-ML corrected the discussion. X-SM and J-SK contributed to the discussion and reviewed the manuscript. All authors contributed to the article and approved the submitted version.

## Funding

The study was supported by Shenzhen Science and Technology Project (JSGG20191129102216637); Shenzhen Basic Research Project (2021300690); Science and Technology Research Project of Liaoning Provincial Department of Education (L202038); Sanming Project of Medicine in Shenzhen (SZZYSM202111002), Shenzhen Science and Technology Program (JCYJ20210324111404012), and Liaoning Provincial Department of Education Project (LJKMZ20221306).

## Acknowledgments

The authors would like to thank all of the rats, the team of investigators, research partners, operations staff involved in this study, and the Key Laboratory of Ministry of Education for Traditional Chinese Medicine Viscera-State Theory and Applications. We want to thank professor Shun-min Li, Xian-sheng Meng, and Jin-song Kuang for scientific and my husband He Tai for help. We thank LetPub (www.letpub.com) and Lisa Owens (St. James’s Hospital) for its linguistic assistance during the preparation of this manuscript.

## Conflict of interest

The authors declare that the research was conducted in the absence of any commercial or financial relationships that could be construed as a potential conflict of interest.

## Publisher’s note

All claims expressed in this article are solely those of the authors and do not necessarily represent those of their affiliated organizations, or those of the publisher, the editors and the reviewers. Any product that may be evaluated in this article, or claim that may be made by its manufacturer, is not guaranteed or endorsed by the publisher.

## Abbreviations

PCOS, Polycystic ovary syndrome; IR, Insulin resistance; TCM, Traditional Chinese medicine; CFDT, Cang-fu-dao-tan; HFD, high-fat diet; CMC, carboxymethyl cellulose; FINS, fasting insulin; FBG, fasting blood glucose; EDTA, ethylene diamine tetraacetic acid; FSH, follicle stimulating hormone; LH, Luteinizing hormone; E_2_, oestradiol; T, testosterone; IL-1β, Interleukin-1β; IL-6, interleukin-6; TNF- α, Tumor necrosis factors- α; CRP, C-reactive protein; ASK1, Apoptosis signal-regulating kinase 1; MAP3K, Mitogen-activated kinase kinase kinase; Mfn1/2, Mitofusin1/2; Drp1, Dynamin-related protein 1; OPA1, Optic atrophy 1; PGC-1 α, PPARγ coactivator-1- α; RT-qPCR, Real-time quantitative Polymerase Chain Reaction; Cyt-c, cytochrome C; DAPI, 4’,6-diamidino-2-phenylindole; DCFH-DA, 2’,7’-Dichlorodihydrofluorescein diacetate; ATP, adenosine triphosphate; RCR, respiratory control rate; MMP, mitochondrial membrane potential; mPTP, mitochondrial permeability transition pore; HPLC, high-performance liquid chromatography; SD, Sprague-Dawley; ANOVA, One-way analysis of variance.
